# “Choose change”: design and methods of an acceptance and commitment therapy effectiveness trial for transdiagnostic treatment-resistant patients

**DOI:** 10.1186/s12888-019-2109-4

**Published:** 2019-06-10

**Authors:** Jeanette Villanueva, Andrea H. Meyer, Marcia T. B. Rinner, Victoria J. Firsching, Charles Benoy, Sandra Brogli, Marc Walter, Klaus Bader, Andrew T. Gloster

**Affiliations:** 1 Department of Psychology, Division of Clinical Psychology and Intervention Science, University of Basel, Basel, Switzerland; 2 Department of Psychology, Division of Clinical Psychology and Epidemiology, University of Basel, Basel, Switzerland; 3 Center for Psychosomatics and Psychotherapy, Psychiatric Hospital of the University of Basel, Basel, Switzerland

**Keywords:** Acceptance and commitment therapy (ACT), Effectiveness, Inpatients, Outpatients, Transdiagnostic, Psychological flexibility, Implementation

## Abstract

**Background:**

Acceptance and Commitment Therapy (ACT) has been successfully established in hundreds of efficacy trials. It is less understood, however, how ACT works in real-world settings. Furthermore, little is known about how contextual variables such as treatment setting (inpatient vs. outpatient), social network and environment of the patient impact outcome.

**Methods:**

This paper describes the methods of the Choose Change study that compares transdiagnostic inpatients (*n* = 85) and outpatients (n = 85) with varying degrees of treatment experience and treatment success (i.e., no previous treatment vs. previous remission vs. treatment-resistant). Patients received ACT during an intensive treatment phase lasting approximately twelve treatment sessions, and were accompanied up to twelve months following intensive treatment. Main outcomes include symptoms, functioning, and well-being. Multiple levels of data are investigated, including treatment context, weekly assessments, a behavioral approach test, multiple follow-up phases, and ambulatory assessment using Event Sampling Methodology, to examine patients’ daily context.

**Discussion:**

We aim to investigate antecedents, consequences, and inherent processes that contribute to the maintenance or fluctuations of psychological disorders and the efficacy of ACT treatment. Furthermore, this study intends to increase understanding of how accurately participants can report on their own experiences, in order to expand our knowledge of how to probe for such information in the future. The results of Choose Change will provide basic clinical theory and clinical care with important and meaningful insights into the effectiveness of ACT, trans diagnostically, in in- and outpatients, and in a naturalistic setting.

**Trial registration:**

This study was retrospectively registered in the ISRCTN Registry (registration number ISRCTN11209732) on May 20th 2016.

## Background

### Acceptance and commitment therapy

Acceptance and commitment therapy (ACT) has been shown to be efficacious in numerous disorders, such as anxiety and depression (e.g., [1]), psychosis (e.g., [2]), phobias (e.g., [3]), alcohol use, anger, and stress (e.g., [4]), obesity (e.g., [5]), burnout [6], and treatment-resistant patients [7]. ACT aims to increase psychological flexibility, which has been shown to increase mental health in over 250 randomized controlled clinical trials across numerous disorders [8]. Psychological flexibility is defined as someone’s ability to “recognize and adapt to various situational demands; shift mindsets or behavioral repertoires when these strategies compromise personal or social functioning; maintain balance among important life domains; and be aware, open, and committed to behaviors that are congruent with deeply held values” ( [9], p865). Preliminary evidence suggests that symptomatology itself does not explain whether someone is functioning with a high level of well-being [10] and there is also evidence that psychologically flexible responses to changing demands promote advantageous outcomes [11]. This speaks to a tendency in basic behavioral science and research away from disorder-specific characteristics and processes, towards processes that potentially underlie multiple disorders and clinical states [12]. However, despite ACT being a transdiagnostic approach [13], there are few transdiagnostic (i.e., applicable across diagnostic categories as opposed to a single disorder) effectiveness studies (e.g., [14, 15]). Further, despite the studies investigating ACT’s efficacy, important questions remain, for example, specification of the mechanisms of action, stability of change, and how well it can be implemented into routine clinical practice [16]. However, in clinical trials, the mechanisms of change are far from clear, and integrated assessment is as necessary as it is difficult to implement. Overall, this study investigates three key aspects: The effectiveness of ACT, long- term follow-up, and the social context and social processes.

### Effectiveness of acceptance and commitment therapy

There is also a need to improve our understanding of the effectiveness of ACT, stressing real-world settings across broad populations with the goal of maximizing external validity [17]. While the importance of Randomized Controlled Trials (RCTs) and efficacy studies is evident, such tightly controlled and randomized studies might neglect crucial factors of what is done in the field [18], and thus less likely reflect conditions under which interventions are used in common clinical practice [19]. Non-randomized trials are often more externally valid than RCTs because they include all patients, are conducted under conditions that more closely match how practitioners will ultimately implement the treatment, and include more context variables (such as patient-practitioner relationship, [20]). Objective and subjective ratings need to be assessed, for instance through paradigms assessing psychological flexibility during a psychological challenge (i.e. a behavioral approach test).

### Long-term follow-up

Relatively little is known about the processes that unfold following treatment, for instance, whether or how patients integrate newly learned skills into their daily environment, and how these factors interrelate with well-being of both the patients and their social networks. Processes following treatment have traditionally been examined with a single assessment concentrating on symptomatology, which is why researchers have been encouraged to include longer follow up periods in clinical studies [21]. The period following treatment is risky if patients stop applying what was learned during therapy or are confronted with challenges [21, 22]. This is especially true for treatment-resistant patients (i.e., patients who do not respond to standard, first line treatments), for whom viable treatment options are lacking and even less is known about than about first-line treatments [7]. To determine if ACT can meaningfully impact treatment-resistant patients in routine practice, a study population including treatment-resistant patients across diagnoses treated in real-world settings is essential. Inclusion of non-treatment-resistant patients, however, is important as well to inform about treatment-resistant ones.

### Social context and social processes

Factors outside therapy itself, including social processes, are believed to account for approximately 33% of improvement in patients undergoing psychotherapy [23]. Despite the importance of social context, it remains poorly understood how the influence of social surroundings (e.g., supportive vs. counterproductive) longitudinally affects the patient’s well-being and prosociality (i.e., cooperating with others, acting for the well-being of others, and sacrificing for others, [24, 25]) and how this in turn affects the patient’s social context. Tracking the transition into the real world after treatment is therefore crucial. The challenges and risks of relapse during this period may be more extreme for inpatients than outpatients, as their change of environment (i.e., leaving the hospital) is more extreme; however, this has not been tested directly. Furthermore, it is completely unknown how the influence of these social variables is moderated by psychological flexibility. Patients treated in an inpatient setting with more negative relationships in their extended social network have been found to relapse more frequently [26]. This suggests that both close and extended social ties are relevant for outcomes, and that these impact multiple forms of treatments. More research is sorely needed to better understand the mechanisms that influence a patient and their social context, particularly in disorders beyond depression and in non-cross-sectional studies that allow the temporal order of effects to be disentangled [27, 28]. The few existing longitudinal studies have identified a bidirectional link between couple distress and depression [27, 28] and to a lesser extent between couple distress and substance abuse [29, 30] and between couple distress and anxiety [30]. Relatively little emphasis has been placed on outcomes of functioning, well-being, or prosocial behaviors – neither in the treated patient nor the immediate and extended social network. As such, little is known about the interaction between treatment, social context, and these broader outcomes.

Building on these insights, the necessity to understand patients’ behavior in their natural environment becomes clear [31]. Daily life happens in specific environmental contexts, and there is a need to understand these contexts. Implementing Event Sampling Methodology (ESM) allows the examination of patients’ daily life and its stressors, including the assessment of moods, thoughts, symptoms or behaviors, which change over time [31, 32]. Thus, ecologically valid data can be collected in a real-time fashion while capturing dynamic changes of variables [33].

### Study aims

The major aim of *Choose Change* is to longitudinally examine the mechanisms of action involved in an ACT treatment for transdiagnostic patients with varying degrees of treatment experience and treatment success in a controlled effectiveness trial in order to maximize external validity [17]. More specifically, it will be tested how psychological flexibility training (i.e., ACT) influences various outcomes across time and document how the intervention is implemented in patients’ everyday lives following the treatment. Simultaneously, state-of-the-art methods are used to ascertain processes of change and maintain internal validity. Increased use of technological innovations, such as mobile phones, will provide us with more information about the common and specific effects of psychological treatments [34]. This is the first project to test the effects of the social context of treatment settings (in- vs. out-patient) and the transition out of intensive treatment to well-being, functionality, and recidivism following a modern transdiagnostic behavioral treatment promoting psychological flexibility.

#### Main research areas

We will investigate the immediate and long-term outcomes of treating treatment-resistant patients with psychological flexibility training (i.e., ACT) in two treatment-related social contexts (i.e., inpatient and outpatient), and the influence of other, naturally occurring social contexts (e.g., self-chosen contexts such as home, work etc.) on various treatment outcomes. These research areas include broad outcomes (i.e., indices of well-being, functioning, social interactions, and prosocial behavior) in addition to symptoms and recidivism, thus going beyond a symptom-based focus.

On the basis of these main research questions, we derived the following main hypotheses: First, we expect the ACT intervention to lead to significant and clinically relevant changes during treatment that remain stable or increase at 12-month follow-up. Second, patients treated in the inpatient setting will experience more intense and frequent social stressors in the follow-up period than those treated in the outpatient setting, which will have a negative impact on outcomes. Third, psychological flexibility will moderate the negative impact of social stress on outcomes over time, so that it is buffered in patients with high levels of psychological flexibility.

## Method

### Design

**Fig. 1:**
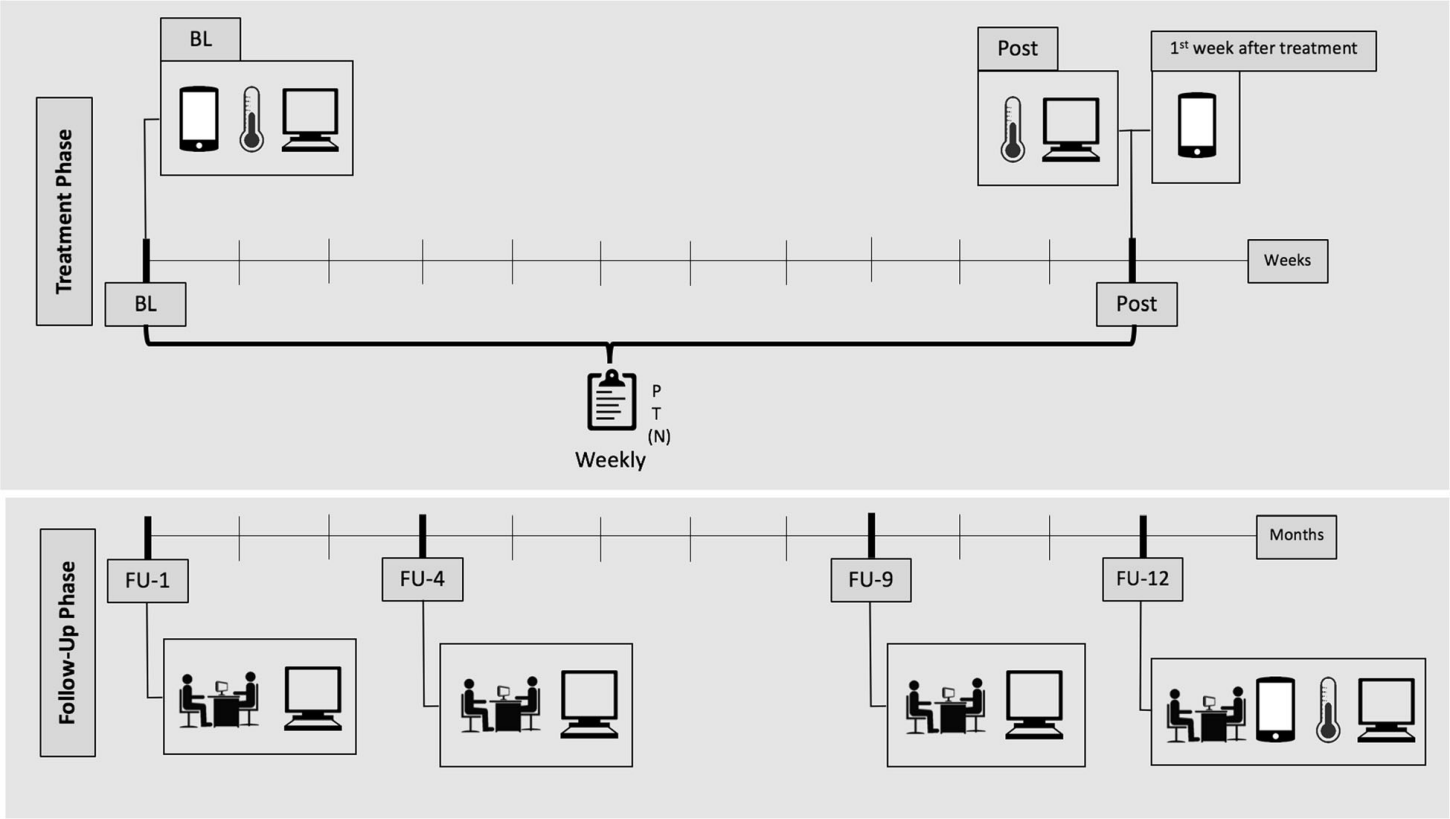
Illustration of the study design. *Note*. Smartphone, Heat Induction Test, Assessment is administered at Baseline, Post, and 12-month follow-up. Process assessment is assessed weekly during the intensive treatment phase by the patient, therapist, and nurse (in the case of the inpatient setting). Assessment through questionnaires and interview are administered at 1-, 4-, and 9-month follow up. BL: Baseline; P: Patient; T: Therapist; N: Nurse (only for the inpatient setting). FU-1, FU-4, FU-9, FU-12: Assessments at 1-, 4-, 9-, and 12-month follow up

This is a controlled effectiveness clinical trial. The study contains multiple seven-day-ESM phases, four intensive follow-up assessments at 1, 4, 9, and 12 months following intensive treatment, weekly measures during treatment, and a behavioral approach test, while considering the effects of the social context of treatment settings (in- vs. outpatient), and following a modern transdiagnostic behavioral treatment promoting psychological flexibility. Figure 1 illustrates an overview of the study design.

### Sample size

Power analyses were conducted with alpha = 0.05, power = 0.8, two-sided test, for within group and between group hypotheses. We assumed a correlation between time points = 0.5 and based on previous work (Gloster et al., 2015), a small to medium effect size was assumed. Required sample sizes were 75 individuals for within group research and 150 patients (75 per group) for between-group hypotheses. Taking missing values into account (ca. 10% dropout), the required sample size in order to have sufficient power for all three hypotheses was thus approximately 170 individuals. We therefore aim to recruit *n* = 85 patients per treatment modality.

### Participant recruitment and selection criteria

Participants are recruited from two specialized clinics practicing ACT (inpatient and outpatient) from ongoing referral and intake procedures. Since ACT is a transdiagnostic therapy which is efficacious for many disorders, and in order to increase generalizability within this effectiveness trial, selection criteria are purposefully kept to a minimum. Inclusion criteria are: Minimum 18 years of age, ability to speak German sufficiently, present for therapy and able to attend sessions, previous treatment (i.e., ≥ 20 sessions of empirically supported psychotherapy and/or minimum dosage and length of an approved drug as recommended by international guidelines), and sign a consent statement will be included the study. In addition, for patients in the out-patient setting, patients will also be included with < 20 sessions therapy experience to enable full modelling of patients from those without treatment experience, to those with treatment experience in the outpatient setting, to those with treatment experience in the inpatient setting. Exclusion criteria include acute suicidal intent, acute substance dependency, (where the primary treatment goal is detoxification), active mania, previous experience with ACT, and inability to read or complete assessments. Otherwise all diagnoses are included (i.e. Affective Disorders, Anxiety Disorders, Somatoform Disorders, Mood Disorders, Anxiety-stress related Disorders, Somatic Disorders, Obsessive-Compulsive Disorder, Impulse Control Disorders, Gambling Disorders, and Personality Disorders). When participants enter the clinic, medication is optimized when necessary, as determined by the attending physician in consideration of patient preference. Medication is then held constant throughout the intensive treatment phase.

### Outcome assessments

Assessments in questionnaire and interview format will occur at six primary time points to capture changes that the patients choose in the following domains: Therapy expectancy, psychological flexibility, social interactions, therapy consistent skills, well-being, emotion regulation, symptomatology, stress, and prosocial behavior. ESM will target the same domains. Table 1 shows a brief description of the assessments. Assessments are done by psychology graduates and doctoral students. Assessors are trained to competency and supervised regularly. All utilized assessments were chosen based on covering the respective domain and adequate quality criteria.

**Table 1:** Description of assessment instruments of the Choose Change study

Abbreviation	Description
CEQ	The **Credibility and Expectancy Questionnaire** [50] is a 6-item scale for measuring treatment expectancy and rationale credibility for use in clinical outcome studies. There are two rating scales, one from 1 to 9 and another (Item 4) from 0 to 100%.
AAQ-II	The **Acceptance and Action Questionnaire** [51] is a 10-item self-report measure of psychological flexibility and acceptance. Each item is rated on a 7-point scale from 1 (never true) to 7 (always true).
PF	The **PsyFlex** [42] is a self-developed 6-item self-report measuring the process of Psychological Flexibility. Each item is rated on a 5-point scale from 1 (very often) to 5 (very rarely).
BAT	The **Behavioral Avoidance Test: Radiant Heat Induction Test** [52]**.** Thermal testing is performed by applying a thermode on the patient’s skin. A patient’s response to temperature stimuli (the sensory thermal threshold) is recorded.
OSS-3	The **Oslo Social Support Scale** [53] is a 3-item self-report measure of social support. Item 1 is rated on a 4-point scale, items 2 and 3 are assessed on a 5-point scale.
SIS	The **Social Interaction Scale** is a self-designed 3-item self-report measuring the frequency, duration, and discomfort of social interactions in the last 24 h. Item one is rated on a 4-point scale, item 2 on a 3-point scale.
RAS	The **Relationship Assessment Scale** [54] is a 7-item self-report measure of relationship satisfaction. Each item is rated on a 7-point scale from 1 (low satisfaction) to 5 (high satisfaction). Items 4 and 7 are reverse scored.
SNQAS	The **Social Network Quality and Action Scale** is a self-developed 10-item scale measuring the relationship quality with important people in a person’s life. In item 1, up to 15 people can be listed, Items 2–10 are assessed individually for every person mentioned in item 1. Items 2 and 3 specify the relation with the person concerned. Of the following items, 4 are rated on a 7-point scale, 2 on a 5-point scale and 1 on a 4-point scale.
CFQ	The **Cognitive Fusion Questionnaire** [55] is a 7 item self-report measure of cognitive fusion. Each item is assessed on a 7-point scale from 1 (never true) to 7 (always true).
FFMQ-SF	The **Five Facet Mindfulness Questionnaire – Short Form** [56] is a 24-item self-report measure of different aspects of mindfulness. Each item is measured on a 5-point scale from 1 (never applies) to 5 (always applies).
VLQ	The **Valued Living Questionnaire** [57] is a 2-item self-report measure assessing valued living in 12 different life domains. Both items are assessed on a 10-point scale from 1 (not important) to 10 (very important).
WHODAS 2	The **World Health Organization Disability Assessment Schedule – 2** [58] is a 17-item self-report disability assessment with 7 domain-specific scores. Each item is rated on a 5-point scale from 1(no difficulty) to 5 (extreme difficulty or cannot do).
MHC-SF	The **Mental Health Continuum – Short Form** [59] is a 14-item self-report measure of emotional, social and psychological well-being. Each item is rated on a 6-point scale from 0 (never) to 5 (every day)
MLQ	The **Meaning in Life Questionnaire** [60] is a 10-item measure of meaning in life. Each item is assessed on a 7-point scale from 1 (absolutely untrue) to 7 (absolutely true).
DERS	The **Difficulties in Emotion Regulation Scale** [61] is a 36-item self-report measure of emotion dysregulation. Each item is rated on a 5-point scale from 1 (almost never) to 5 (almost always).
ASQ	The **Affective Style Questionnaire** [62] is a 20-item self-report measure of individual differences in emotion regulation. Each item is rated on a 5-point scale from 1 (not true of me at all) to 5 (extremely true of me).
SCID-I/Screener	The **Structured Clinical Interview for DSM-IV – Axis I disorders** [38] is a semi-structured interview guide that enables mental health professionals to make DSM-IV Axis I diagnoses.
Severity Scale	The **Anxiety Disorders Interview Schedule (ADIS) severity rating scale** [39] was utilized to rate the diagnoses on a scale from 0 (absent) to 8 (very severe)
BDI-SF	The **Beck Depression Inventory – Short Form** [63] is a 7-item self-report measuring severity of symptoms associated with depression. Each item is rated on a 4-point scale.
BSCL	The **Brief Symptom Checklist** [64] is a 53-item self-report measure. Each item is rated on a 5-point scale from 0 (not at all) to 4 (very strongly).
PSWQ	The **Penn State Worry Questionnaire** [65] is a 16-item self-report assessing the trait of worry. Each item is rated on a 5-point scale from 1 (not a tall typical of me) to 5 (very typical of me).
FQ	The **Fear Questionnaire** [66] is a 24-item self-report of phobia. Each item is rated on a scale from 0 to 8.
OCI-R	The **Obsessive-Compulsive Inventory – Revised** [67] is an 18-item self-report measure of obsessive-compulsive symptoms. Each item is rated on a point scale from 0 (not at all) to 4 (extremely).
SIAS	The **Social Interaction Anxiety Scale** [68] is a 20-item self-report measure of fears in general social interaction. Each item is rated on a 5-point scale from 0 (not at all) to 4 (extremely).
PSS	The **Perceived Stress Scale** [69] is a 14-item self-report measure of perceived stress in different situations. Each item is rated on a 5-point scale from 0 (never) to 4 (very often).
DSS	The **Daily Stress Scale** [70] is a 7-item self-report measure of perceived stress in different life domains. Each item is rated on a 7-point scale from 1 (little incriminating) to 7 (not affected)
DAP-PS	The **Developmental Assets Profile – Prosocial Subscale** [71] is an 8-item self-report measure of developmental assets linked to different indicators of well-being. Each item is rated on a 4-point scale from 1 (not at all or rarely) to 4 (extremely or almost always)
EVOS	The **Prosociality questionnaire** is a self-designed 9-item self-report asking about prosociality in partnerships and derived from prosocial principles [72]. Items 1, 6, 7, and 9 are rated on a 5-point scale. Items 2–5, and Item 8 are rated on a 3-point scale.
COOP	The **Cooperation Scale** [73] is a 13-item self-report measure of cooperation in social situations. Each item is rated on a 6-point scale from 1 (I don’t agree at all) to 6 (I totally agree).
SRAS	The **Self Report Altruism Scale** [74] is a 20-item self-report measure of altruistic behavior. Each item is rated on a 5-point scale from 1 (never) to 5 (very often).

*Note.* Full names of the instruments are printed in bold for a better overview

Primary outcome measures will target symptoms and general functioning. These are assessed using questionnaires completed by the patients using the Brief Symptom Checklist (BSCL; to assess symptoms), the World Health Organization Disability Assessment Schedule – 2 (WHODAS 2; to assess functioning), and the Mental Health Continuum – Short Form (MHC-SF; to assess well-being). Primary outcomes are measured at baseline, post, and 12 months following treatment. The MHC-SF is measured at all time points.

Secondary outcomes are included to assess additional domains, such as treatment context, social context, spreading of effects, follow-up, daily context, and behavioral measures (see Table 2 and Table 3). In addition, an experimental paradigm will be used to measure how psychological flexibility impacts patients’ behavioral response to experimentally induced discomfort (i.e., radiant heat pain). This radiant heat pain paradigm is commonly used in research [35, 36] and will be administered at baseline, post, and 12 months after treatment.

**Table 2:** Overview of domains, constructs, assessments, and respective time points at which each assessment is administered

Choose Change: Assessment Overview								
Domain	Construct	Instrument/Paradigm	Assessment Time					
			BL	Post	FU-1	FU-4	FU-9	FU-12
Therapy Expectancy	Expectancy	Credibility and Expectancy Questionnaire (CEQ)	x					
Psychological Flexibility	Psychological Flexibility/ Emotional Avoidance	Acceptance and Action Questionnaire – II (AAQ-II)	x	x				x
	Process of Psychological Flexibility	PsyFlex (PF)	x	x	x	x	x	x
	Behavioral Test/Experiment	Behavioral Avoidance Test (BAT): Heat Induction Test	x	x				x
Social interactions	Overall Social Support	Oslo Social Support Scale (OSS-3)	x	x				x
	Social Interaction	Social Interaction Scale (SIS)	x^i^	x^i^	x	x	x	x^i^
	Relationship Satisfaction	Relationship Assessment Scale (RAS)	x^i^	x^i^		x		x^i^
	Social Network Quality	Social Network Quality and Action Scale (SNQAS)	x^i^	x^i^	x	x	x	x^i^
ACT-Skills	Cognitive Fusion	Cognitive Fusion Questionnaire (CFQ)	x	x				x
	Mindfulness	Five Facet Mindfulness Questionnaire - short version (FFMQ-FS)	x	x				x
	Values	Valued Living Questionnaire (VLQ)	x	x				x
Well-being	General Functioning	WHO Disability Assessment Schedule 2.0 (WHODAS 2)	x^p^	x				x
	Flourishing Mental Health/ well-being	Mental Health Continuum – Short Form (MHC-SF)	x^ip^	x^i^	x^i^	x^i^	x^i^	x^i^
	Meaning of Life	Meaning in Life Questionnaire (MLQ)	x	x				x
Emotion Regulation	Problematic Emotion Regulation	Difficulties in Emotion Regulation Scale (DERS)	x	x				x
	Emotion Regulation	Affective Style Questionnaire (ASQ)	x	x				x
Diagnosis/ Symptomatology	DSM Diagnostic Information	Structured Clinical Interview for DSM-IV (SCID-I/Screener)	x					
	Depression	Beck Depression Inventory – Fast Screen (BDI-FS)	x	x	x	x	x	x
	Problems and Discomfort	Brief Symptom Checklist (BSCL)	x^p^	x				x
	Worry (GAD)	Penn State Worry Questionnaire (PSWQ)	(x)	(x)		(x)		(x)
	Anxiety/Fear	Fear Questionnaire (FQ)	(x)	(x)		(x)		(x)
	Obsessions and Compulsions (OCD)	The Obsessive-Compulsive Inventory, short Version (OCI-R)	(x)	(x)		(x)		(x)
	Social Fear	Social Interaction Anxiety Scale (SIAS)	(x)	(x)		(x)		(x)
Stress	Perceived stress	Perceived Stress Scale (PSS)	x	x				x
	Daily stress	Daily Stress Scale (DSS)	x^i^	x^i^	x	x	x	x^i^
Prosocial Behavior	Prosocial Behaviors	Developmental Assets Profile – Prosocial Subscale (DAP-PS)	x	x				x
		Prosociality Questionnaire (EVOS)	x	x				x
	Cooperation	Cooperation Scale (COOP)	x	x				x
		The Self-Report Altruism Scale (SRAS)	x	x				x
Therapy Consistent Skills	Transfer of Skills to Daily Life	Vignettes			x	x	x	x

*Note.* BL = Baseline; FU-1, FU-4, FU-9, FU-12 = Assessments at 1-, 4-, 9-, and 12-month follow up; ESM = Event Sampling Methodology (smartphone); i = to be completed by close person in addition to patient; () = to be filled out only by patients who have that specific symptomatology (SCID); p = primary outcome measures

In order to test whether treatment context has an impact on patients’ well-being and functioning (i.e. flourishing) [37], ACT will be administered for both in- and outpatients. For inpatients, a multidisciplinary team is involved, consisting of psychotherapists providing individual and group therapy and the nursing and support staff providing ACT specialized sessions. For outpatients, psychotherapists provide individual ACT sessions. All psychotherapists are certified for this study through attendance of several ACT trainings and provision of a certification role play video rated by a certified ACT trainer. Therapists will make choices based on ACT skills and procedures, which will all be documented following each session. This allows for the documentation of ACT’s clearly defined skills, while enabling a flexible and situationally sensitive application.

#### Baseline: diagnostics, assessment

During the first week of therapy participants complete informed consent procedures before data collection. All participants complete the Structured Clinical Interview for DSM-IV Axis I Disorders (SCID) [38] to determine diagnostic status. Diagnoses are rated on the Anxiety Disorders Interview Schedule (ADIS) severity rating scale [39]. The diagnosis with the highest severity score determines the primary diagnosis. Following the SCID, participants complete diagnostic-specific and transdiagnostic questionnaires (see Table 2).

#### Post: assessment

At completion of the intensive therapy phase post assessment is done (i.e. diagnostic-specific and transdiagnostic questionnaires, ESM, and the heat induction test). Except for the SCID and the Credibility and Expectancy Questionnaire (CEQ) procedures and items will be the same as in baseline (see Table 2 and Table 3).

**Table 3:** Overview of domains and respective time points at which each EMA assessment is administered

Choose Change: Assessment Overview			
Domains assessed	Assessment Time for ESM		
	BL	Post	FU-12
Therapy Expectancy			
Psychological Flexibility	x	x	x
Social interactions	x	x	x
ACT-Skills	x	x	x
Well-being	x	x	x
Emotion Regulation	x	x	x
Diagnosis/ Symptomatology	x	x	x
Stress	x	x	x
Prosocial Behavior	x	x	x
Therapy Consistent Skills			

*Note. BL* Baseline, *ESM* Event Sampling Methodology (smartphone), *FU-12* Assessments at 12-month follow up

#### Follow-up: 1, 4, 9, and 12 months after treatment

In order to assess what was learned during therapy and how psychological flexibility, well-being, and symptoms are associated with each other, patients stay in contact with the research team after completing the intensive inpatient or outpatient treatment phase. Over the following year, four follow-up appointments are conducted at 1, 4, 9, and 12 months after completion of the intensive therapy phase. Follow-up appointments include an online questionnaire and an interview. For the included questionnaires see Table 2.

### Assessments measuring mechanisms of action across levels of analysis

#### Behavioral approach test

A heat induction test (using the TSA-II, [40]) was used in order to test how participants respond to an uncomfortable and ambiguous stimulus not subject to the same demand characteristics as questionnaires. During this test, temperature increases until the patient stops the increase with a click of the mouse. This temperature is then held constant to test tolerance. This test is administered at Baseline and Post (to determine whether the tolerance changes during treatment) and at Follow-Up 12 (to determine whether the tolerance changes after treatment). Before and after participants will complete a pain-related questionnaire.

#### Weekly psychological flexibility process measure (between baseline and post)

In order to examine whether patients with higher levels of psychological flexibility will profit faster from treatment, weekly psychological flexibility progression will be measured during the intensive treatment phase (between Baseline and Post). For this, the PsyFlex [41, 42] will be used across different information sources. The PsyFlex is a self-developed instrument which aims at measuring state psychological flexibility through capturing all six skills of the ACT Hexaflex [43]. The PsyFlex is filled out by the patients, their therapists, and – in the inpatient setting – their nurse, which results in an evaluation of one person from up to three different perspectives.

#### Ambulatory monitoring (ESM at baseline, post, and 12 months after treatment)

In order to examine how psychological flexibility and other treatment parameters were applied to stressors in real-time in the participants’ natural environment, how social group interactions affected the individual, and how these aspects changed over time, patients receive a smartphone from the research team and instructions on its usage (i.e. operation of the smartphone, recognition of the signaling tone, answer example questions). Data will be collected at Baseline, Post, and Follow-Up 12. At each time point, patients will carry the smartphone for one week, using a signal-contingent ESM approximately every three hours (e.g., 8 am, 11 am, 2 pm, 5 pm, 8 pm, and 11 pm). Items stem from previous ESM studies [44–47] and are based on a functional analysis of social interactions [48] due to their individual nature. In addition to the data assessed via questionnaires, GPS data (e.g. time-stamped data regarding location, speed of travel if travelling, and time spent at different locations) of the patients will be collected during the respective ESM week. For assessed domains within the ambulatory assessement, see Table 3.

#### Close person (baseline, post, and 12 months after treatment)

In order to investigate the impact of the social context, patients are asked to give the research team permission to contact a person of their choice, who is close to them. This close person is then contacted by the research team. After obtaining informed consent from this close person, an online questionnaire is completed at Baseline, Post, and at Follow-Up 12 by the close person, providing information about their relationship and well-being. For the included questionnaires see Table 2.

#### Therapy integrity

Therapy will consist of an intensive treatment phase lasting approximately twelve treatment sessions. Therapists will be certified for this study. Therapy sessions will be recorded and of those, two will be randomly selected and rated by external and independent ACT experts to assess treatment integrity. Therapy integrity will be further promoted via regular targeted supervision sessions by ACT experts.

### Analysis plan

For all analyses we will use multilevel models with time as level 1 and person as level 2 variables. These models are able to deal with the interdependence of observations across time within the same subject. Hypothesis 1 refers to a multilevel model with time as within-subjects variable and no between-subjects variable. Hypothesis 2 is based on hypothesis 1 but will contain in addition one between-subjects factor (group, 2 levels), plus the interaction between time and group, which is our primary focus. Hypothesis 3 refers to a multilevel model with social stress as predictor and psychological flexibility as mediator. Again, we will be interested in the interaction between these two characteristics, which are both time-varying within subjects.

## Discussion

The aim of this study is to longitudinally examine the mechanisms of action involved in an Acceptance and Commitment Therapy (ACT) treatment for transdiagnostic patients with varying degrees of treatment experience in a controlled effectiveness trial. This study captures the treatment effectiveness of ACT, in addition to assessing psychological flexibility and patients’ social contexts in multiple ways over multiple time points, up to 12 months after intensive in- or outpatient treatment. A large sample consisting of patients with various diagnoses is recruited, in accordance with ACT being a transdiagnostic behavioral approach. Novel methods of data collection (Event Sampling Methodology, ESM) are combined with traditionally used ones such as questionnaires (implemented at many time points in many ways) and a behavioral approach test (Heat Induction Test). By capturing treatment effectiveness (including follow-up to 12 months), symptoms, emotions, social interactions, stressors, psychological flexibility, spreading of effects to the patient’s social network, well-being, and health-behaviors as well as behavioral variables, and fluctuations of all these, this study will add to the understanding of antecedents, consequences, and inherent processes that contribute to the recovery of psychological disorders and maintenance of gains as well as fluctuations in well-being, functioning and reduced symptomatology. Furthermore, these data allow for a better understanding how time affects the accuracy of participants’ reporting of their own experiences, thereby contributing to a better understanding of how to better probe for such information in the future [49]. Therefore, these data are relevant for both basic clinical theory and clinical care.

The implementation of ESM enables us to capture experiences in participants’ natural environment. ESM is also today’s gold standard and allows us to collect information closer to real life than data collection solely with questionnaires. Since participants’ retrospective recalls are limited to three hours during the ESM week, the participants will have more accurate memories regarding that time window than larger time windows (days, weeks, months, or even years) often enquired about in questionnaires. Combining ESM, behavioral tests, weekly process measures filled out by three sources (patient, therapist, and nurse [in the in-patient setting]), questionnaires, and also information about the participants’ social networks in a large group of in- or outpatients with various diagnoses across different severity levels results in a unique and rich data set.

These data further the understanding of symptoms, general functioning, and well-being by testing across different domains, namely therapy expectancy, psychological flexibility, social interactions, therapy consistent skills, well-being, emotion regulation, symptomatology, stress, and prosocial behavior. They will also provide information about how ACT differentially affects a range of psychological, behavioral, cognitive, and affective outcomes. Different components can be identified and related to symptomatology, maintenance of therapeutic gains, and relapse.

Finally, Choose Change will generate hypotheses that will lead to a number of publications that will focus on how ACT transfers to treatment-resistant patients treated in in- and outpatient treatment settings. Moreover, the mechanisms underlying treatment success and failure remain largely unknown and findings from Choose Change are expected to significantly contribute to our understanding in this area. The results of Choose Change will provide science with important and meaningful insights into the effectiveness of ACT, trans diagnostically, in in- and outpatients, and in a naturalistic setting.

## Abbreviations

AAQ-II: Acceptance and Action Questionnaire
ACT: Acceptance and Commitment Therapy
ADIS: Anxiety Disorders Interview Schedule
ASQ: Affective style questionnaire
BAT: Behavioral Avoidance Test: Radiant Heat Induction Test
BDI-SF: Beck Depression Inventory – Short Form
BSCL: Brief Symptom Checklist
CEQ: Credibility and Expectancy Questionnaire
CFQ: Cognitive Fusion Questionnaire
COOP: Cooperation Scale
DAP-PS: Developmental Assets Profile – Prosocial Subscale
DERS: Difficulties in Emotion Regulation Scale
DSS: Daily Stress Scale
ESM: Event Sampling Methodology
EVOS: Prosociality questionnaire
FFMQ-SF: Five Facet Mindfulness Questionnaire – Short Form
FQ: Fear Questionnaire
GPS: Global Positioning System
MHC-SF: Mental Health Continuum – Short Form
MLQ: Meaning in Life Questionnaire
OCI-R: Obsessive-Compulsive Inventory – Revised
OSS-3: Oslo Social Support Scale
PF: PsyFlex
PSS: Perceived Stress Scale
PSWQ: Penn State Worry Questionnaire
RAS: Relationship Assessment Scale
RCT: Randomized Controlled Trials
SCID-I: Structured Clinical Interview for DSM-IV – Axis I disorders
SIAS: Social Interaction Anxiety Scale
SIS: Social Interaction Scale
SNQAS: Social Network Quality and Action Scale
SRAS: Self Report Altruism Scale
TSA-II: NeuroSensory Analyzer
VLQ: Valued Living Questionnaire
WHODAS 2: World Health Organization Disability Assessment Schedule – 2
